# Preparation and Characterization of Blank and Nerolidol-Loaded Chitosan–Alginate Nanoparticles

**DOI:** 10.3390/nano12071183

**Published:** 2022-04-01

**Authors:** Rahaf M. Ahmad, Yaser E. Greish, Hesham F. El-Maghraby, Loay Lubbad, Yahia Makableh, Fayez T. Hammad

**Affiliations:** 1 Department of Surgery, College of Medicine & Health Sciences, United Arab Emirates University, Al Ain P.O. Box 17666, United Arab Emirates or rmahmad19@nano.just.edu.jo (R.M.A.); Loay_Lubbad@uaeu.ac.ae (L.L.); 2 Institute of Nanotechnology, Jordan University of Science and Technology, Irbid 22110, Jordan; yfmakableh@just.edu.jo; 3 Department of Chemistry, College of Science, United Arab Emirates University, Al Ain P.O. Box 17666, United Arab Emirates; y.afifi@uaeu.ac.ae (Y.E.G.); habdelrehim@uaeu.ac.ae (H.F.E.-M.); 4 Department of Ceramics, National Research Centre, NRC, Cairo 12622, Egypt

**Keywords:** chitosan, alginate, nanoparticles, nerolidol, hydrophobic

## Abstract

Recently, there has been a growing interest in using natural products as treatment alternatives in several diseases. Nerolidol is a natural product which has been shown to have protective effects in several conditions. The low water solubility of nerolidol and many other natural products limits their delivery to the body. In this research, a drug delivery system composed of alginate and chitosan was fabricated and loaded with nerolidol to enhance its water solubility. The chitosan–alginate nanoparticles were fabricated using a new method including the tween 80 pre-gelation, followed by poly-ionic crosslinking between chitosan negative and alginate positive groups. Several characterization techniques were used to validate the fabricated nanoparticles. The molecular interactions between the chitosan, alginate, and nerolidol molecules were confirmed using the Fourier transform infrared spectroscopy. The ultraviolet spectroscopy showed an absorbance peak of the blank nanoparticles at 200 nm and for the pure nerolidol at 280 nm. Using both scanning and transmission electron microscopy, the nanoparticles were found to be spherical in shape with an average size of 12 nm and 35 nm for the blank chitosan–alginate nanoparticles and the nerolidol-loaded chitosan–alginate nanoparticles, respectively. The nanoparticles were also shown to have a loading capacity of 51.7% and an encapsulation efficiency of 87%. A controlled release profile of the loaded drug for up to 28 h using an in vitro model was also observed, which is more efficient than the free form of nerolidol. In conclusion, chitosan–alginate nanoparticles and nerolidol loaded chitosan–alginate nanoparticles were successfully fabricated and characterized to show potential encapsulation and delivery using an in vitro model.

## 1. Introduction

The sesquiterpene alcohol nerolidol, also called peruviol, occurs naturally in the essential oils of several plants with floral odors including *Baccharis dracunculifolia* [1,2,3]. Several reports showed that it has protective effects in different conditions and organs such as the heart and kidney [4,5,6]. For instance, nerolidol has been shown to have anti-ulcer [1], anti-tumor [7,8], and skin permeation enhancing effects [9,10]. In addition, it has anti-inflammatory [11,12], anti-trypanosomal [13,14], anti-fungal [15,16], and anti-malarial [17,18] effects.

**Figure 1:**
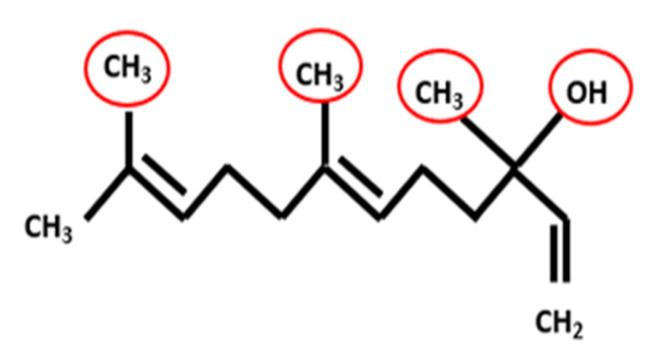
The chemical structure and the functional groups of trans-nerolidol.

Nerolidol (3,7,11–trimethyl-1, 6, 10-dodecatrien-3-ol), has methyl groups at the carbon positions 3, 7, and 11 and a hydroxy group at position 3 (Figure 1). Chemically, two geometric isomers of nerolidol are available: trans and cis forms. It is a farnesane sesquiterpenoid, a tertiary allylic alcohol, and a volatile organic compound [2,3]. Similar to other sesquiterpenes, nerolidol has a high hydrophobicity which results in a poor oral bioavailability, a lack of dose proportionality, and failure to achieve steady-state plasma concentration [19]. Therefore, using nanoparticles to facilitate the delivery and to increase the bioavailability of such agents might be a promising strategy [20,21].

Sodium alginate is a biodegradable, non-toxic polymer that has been used widely in drug delivery [22]. It is a plant-originated polysaccharide which consists of D-mannuronic and -L-guluronic acid residues linked by [1,2,3,4] glycosidic bonds. The use of alginate in a controlled release formulation was shown to produce less vertical mobility of the active ingredient in comparison with the pure product [23]. However, alginate nanoparticles are not stable at room temperature [24] and the encapsulated hydrophobic active compounds leak easily from the nanoparticles. These limitations can be avoided by ionic crosslinking of the anionic alginate nanoparticles with a cationic polymer such as chitosan, which is a cationic, animal-originated polysaccharide produced by the deacetylation of chitin [24,25]. Alginate-chitosan nanoparticles were previously used to encapsulate and efficiently deliver nifedipine [20], doxorubicin [26], and many other agents. The use of alginate and chitosan as a drug delivery system for nerolidol has not been investigated previously. In the current research, both sodium alginate and chitosan were used in fabricating nerolidol-loaded polymeric nanoparticles.

## 2. Materials and Methods

The following materials and chemicals were used in the synthesis of the blank and nerolidol-loaded nanoparticles: nerolidol (98% purity, 870,000 µg in 1 mL), chitosan (deacetylated chitin, low molecular weight), sodium alginate (high molecular weight with a ratio of mannuronic acid to guluronic acid of 1.56), tween 80 (polyoxyethylene sorbitan monooleate), acetic acid, sodium hydroxide. and hydrochloric acid. All materials were purchased from Sigma-Aldrich.

### 2.1. Fabrication of Blank Chitosan–Alginate Nanoparticles

Chitosan–alginate nanoparticles were prepared by the new pre-gelation of sodium alginate with tween 80 followed by the crosslinking with chitosan. Initially, 40 mg of sodium alginate was dissolved in 40 mL of distilled water, 0.02% *w*/*v* tween 80 was added to the sodium alginate and stirred overnight, and the pH was adjusted to 4.8 using hydrochloric acid. Chitosan (80 mg) was then dissolved in 40 mL of 1% *v*/*v* acetic acid, the solution’s temperature was maintained at 60 °C while stirring for 2 h at high-speed using a magnetic rod and stirrer, and the pH of the solution was adjusted to 5 using sodium hydroxide. Using a syringe and a syringe pump, the chitosan solution was added to the alginate solution in a dropwise manner while stirring at a high speed for 1 h. The formed solution was kept in the fridge overnight to facilitate the formation of nanoparticles with a uniform size and shape. The formed pellet was centrifuged and washed three times with distilled water before any characterization.

### 2.2. Fabrication of Nerolidol-Loaded Chitosan–Alginate Nanoparticles

A nano emulsion using tween 80 as a surface-active agent was formed before the formation of the loaded nanoparticles. Briefly, 200 µL of tween 80 was added to 5 mL of Milli-Q water and stirred for 5 min; while stirring, 200 µL of nerolidol was added drop by drop to the solution and stirred for another 10 min. The emulsion was sonicated (Branson, MO, USA) for 40 min. Chitosan–alginate nanoparticles were prepared by the pre-gelation of sodium alginate with tween 80 followed by the crosslinking with chitosan. First, 40 mg of sodium alginate was dissolved in 40 mL of distilled water; the solution was stirred for 5 min and the pH was adjusted to 4.8 using hydrochloric acid. The nano emulsion was added to the alginate solution drop by drop and stirred for 1 h. Chitosan (80 mg) was then dissolved in 40 mL of 1% acetic acid, the solution’s temperature was maintained at 60 °C while stirring for 2 h at a high speed using a magnetic rod and stirrer, and the pH of the solution was adjusted to 5 using sodium hydroxide. Using a syringe and a syringe pump, the chitosan solution was added to the alginate solution in a dropwise manner while stirring at high speed for 1 h. The formed solution was kept in the fridge overnight to facilitate the formation of uniform size and shape nanoparticles. The formed pellet was centrifuged and washed three times with distilled water before any characterization.

### 2.3. Characterization Techniques

#### 2.3.1. Fourier Transform Infrared Spectrometry

A Fourier transform infrared (FTIR) spectrometer (Shimadzu Fourier Transform Infrared Spectrophotometer, IRPrestige-21, Shimadzu, Kyoto, Japan) was employed to characterize the potential interactions of the polymers in the nanoparticles and the polymers with the drug. The sample was dried to form a thin film before characterization. The FTIR bands were collected between 4000 and 650 cm^−1^ with a resolution of 2 cm^−1^ using an FTIR spectrometer.

#### 2.3.2. Ultraviolet-Visible Spectroscopy

The ultraviolet-visible (UV-Vis) (NanoDrop 2000/c) absorption spectra of the chitosan, alginate, nerolidol-loaded nanoparticles, and blank nanoparticles were recorded. Each sample was dissolved in distilled water at room temperature. The solutions were scanned from 450 to 190 nm to obtain the UV-Vis absorption spectra.

#### 2.3.3. Scanning Electron Microscopy

The nanoparticles were observed using a scanning electron microscope (SEM) (FESEM Quatro S, Thermo Fisher Scientific, Brno s.r.o., Czech Republic). After centrifuging the nanoparticles suspension and removing the supernatant, the pellet was redispersed in distilled water and sonicated for 10 min. Using the drop-by-drop method, the sample was dried to form a thin film. The film was then deposited on the aluminum wafer and coated with carbon for examination.

#### 2.3.4. Transmission Electron Microscopy

The nanoparticles were also observed using a transmission electron microscope (TEM) (PhilipsCM10, Amsterdam, The Netherlands). After centrifuging the nanoparticles suspension and removing the supernatant, the pellet was redispersed in distilled water and sonicated for 10 min. One drop of the nanoparticles suspension was deposited on the grid and examined using TEM.

#### 2.3.5. In Vitro Release for Oral Absorption

The drug release study was performed using the dialysis method apparatus. In short, the total volume of the prepared sample was 85 mL containing 200 µL (174,000 µg) of nerolidol, 3.27 mL (6700 µg) of the prepared nerolidol-loaded nanoparticles was centrifuged, and the pellet was re-dispersed in 1 mL phosphate buffer saline (PBS) solution (pH 7.4). This suspension was filled in a dialysis bag (Cutoff 2000 KD) and the bag was placed in 100 mL PBS solution (pH 7.4). The time-dependent release study was conducted at time intervals of 0, 2, 4, 8, 12, 16, 20, 24, 28, 32, 36, and 48 h. The set up was kept at 37 °C under gentle stirring (100 rpm). As nerolidol has no data about the UV-spectroscopy absorbance, a standard calibration curve of nerolidol concentrations was determined using Equation (1). At the periodic intervals, samples of the release medium were taken out and spectrophotometrically assayed for nerolidol released, and the volume was replaced with fresh dissolution medium.

The concentration of nerolidol was calculated using Equation (1):

(1) y=4.5788x+0.0279

where *y* is the concentration of nerolidol and *x* is the absorbance found for each aliquot.

The encapsulation efficiency was calculated using the following equation:

(2) $$EE\;\%=\left(\frac{Maximum\;Conc.\;Released}{Total\;Concentration}\right)*100\%$$

The release percentage for each assayed sample was calculated using Equation (3):

(3) $$\%\;Release=\left(\frac{Conc.\;Released}{Total\;Concentration}\;\right)*100\%$$

The loading capacity of the nanoparticles with nerolidol was calculated using Equation (4):

(4) $$LC\;\%\;=\left(\frac{wieght\;of\;released\;drug}{total\;wieght\;of\;sample}\right)*100\%\;$$

## 3. Results and Discussion 

### 3.1. Fourier Transform Infrared Spectrometry

**Figure 2:**
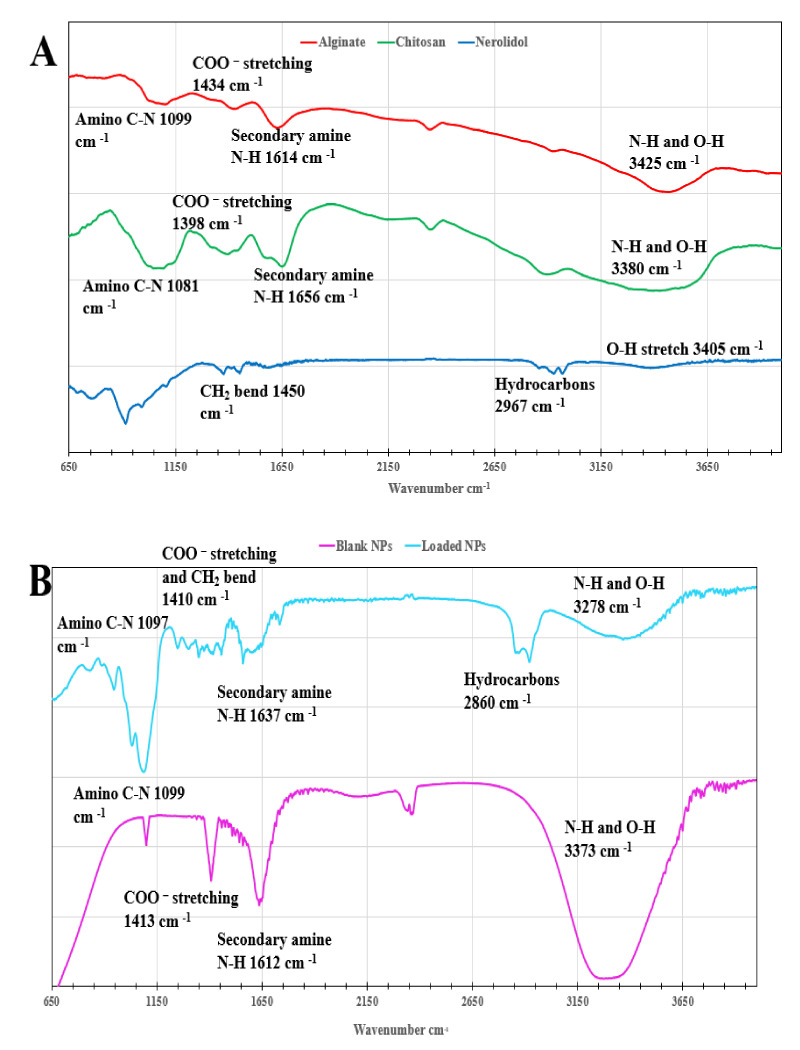
(**A**) The Fourier transform infrared spectrometry (FTIR) of the starting materials and (**B**) the band shifts in the blank and nerolidol-loaded chitosan-alginate nanoparticles.

The FTIR spectra of the blank chitosan–alginate nanoparticles (unloaded), the nerolidol-loaded chitosan–alginate nanoparticles, and the reference chitosan–alginate nanoparticles are shown in Figure 2. The main characteristic peaks of the synthesized blank chitosan–alginate nanoparticles were observed at 1099, 1413, 1612, and 3373 cm^−1^ for the amino group (C-N), carboxylate group (COO^−^), secondary amine (NH_2_), and the amine and hydroxyl groups stretch (N-H and O-H), respectively. Furthermore, in the synthesized nerolidol-loaded chitosan–alginate nanoparticles, the main characteristic peaks were observed at 1097, 1410, 1637, and 3373 cm^−1^ for the amino group (C-N) and the CH_2_ bend, carboxylate group (COO^−^), the secondary amine group (NH_2_), and the amine and hydroxyl (N-H and O-H) stretch, respectively. Regarding the nerolidol hydrocarbon chain and the alcoholic hydroxyl (O-H) stretch, the peak was observed at 2860 and 3278 cm^−1^, respectively. In comparison with the starting materials’ FTIR spectra (Figure 2A), the characteristic peaks of chitosan were observed at 1081, 1398, 1656, and 3380 cm^−1^ for the amino group (C-N), carboxylate group (COO^−^), secondary amine (NH_2_), and the amine and hydroxyl groups stretch (N-H and O-H) [27], respectively. The characteristic peaks of alginate were observed at 1099, 1434, 1614, and 3425 cm^−1^ for the amino group (C-N), carboxylate group (COO^−^), secondary amine (NH_2_), and the amine and hydroxyl groups stretch (N-H and O-H) [27], respectively. Finally, regarding the nerolidol CH_2_ bend, the hydrocarbon chain, and the hydroxyl stretch (O-H), the peaks were observed at 1450, 2967, and 3405 cm^−1^, respectively.

Correspondingly, in the chitosan–alginate nanoparticles and the nerolidol-loaded chitosan–alginate nanoparticles, the peak absorbance of the amino groups, carboxyl groups, secondary amine groups, and the amine and hydroxyl stretching vibrations showed a minor shift following complexation. These results indicate that the carboxylate group of the alginate successfully interacted with the protonated chitosan amino group by electrostatic attraction. Additionally, in the nerolidol-loaded chitosan–alginate nanoparticles, the alcoholic hydroxyl stretch and the symmetric hydrocarbons band showed a minor shift. These band shifts confirm the presence of an interaction between the nanoparticles complex and the nerolidol.

### 3.2. Ultraviolet-Visible Spectroscopy

**Figure 3:**
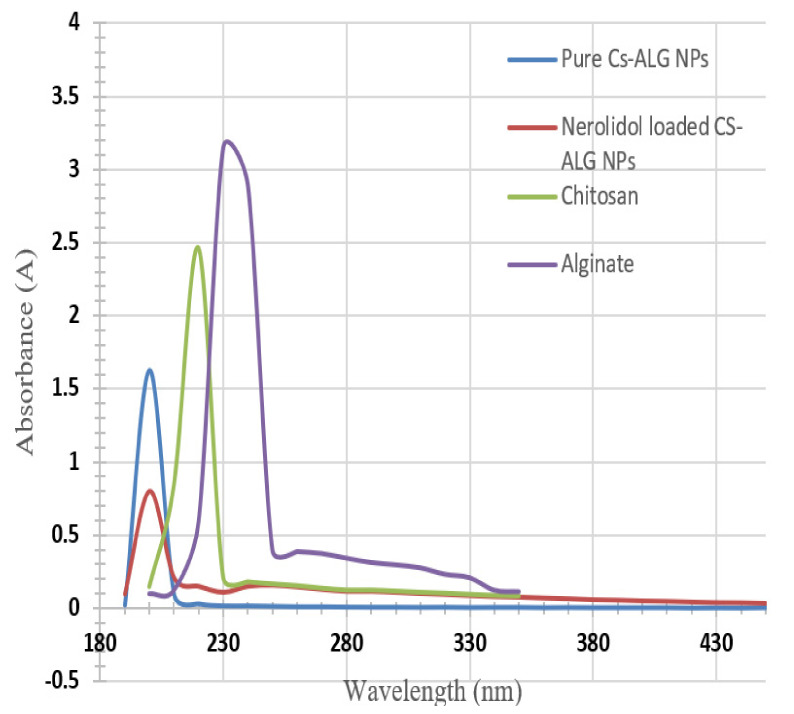
The UV-spectroscopy graphs for chitosan, alginate, blank, and nerolidol-loaded chitosan-alginate nanoparticles.

**Figure 4:**
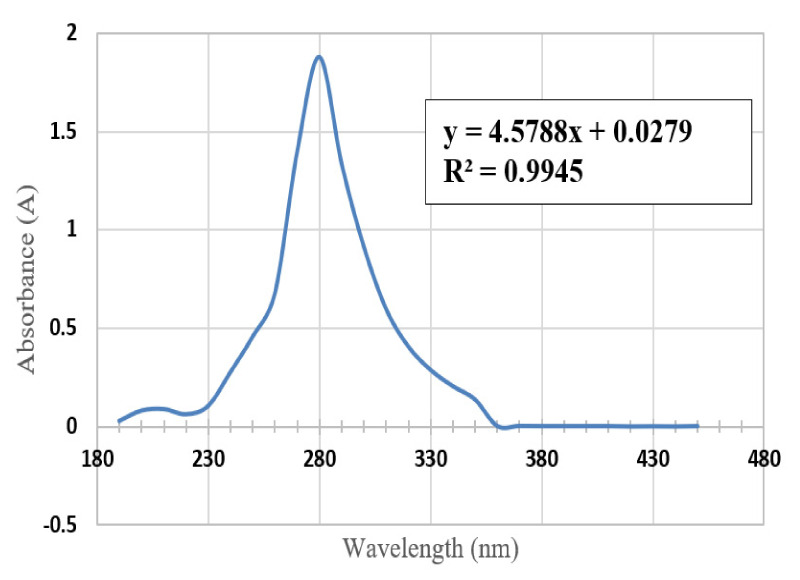
The absorbance peak of diluted nerolidol.

The absorption peak of the chitosan, alginate, and blank chitosan–alginate nanoparticles were observed at 220, 230, and 200 nm, respectively, as shown in Figure 3. The absorption peak of nerolidol-loaded chitosan–alginate nanoparticles was observed as a broad peak between 230 and 280 nm, in addition to the nanoparticles peak at 200 nm as shown in Figure 3. Furthermore, when the pure nerolidol sample was diluted with ethanol, its absorption peak was observed at 280 nm as shown in Figure 4. In the current study, nerolidol was always analyzed in a diluted form. The wavelength value of 280 nm was used to construct the calibration curve of nerolidol and to obtain Equation (1), which is shown in Figure 4.

Comparing the wavelength of the chitosan and alginate to the nanoparticles’ wavelength in the current study (Figure 3), the shift in the absorption wavelength peak was due to the binding between alginate and chitosan in both the blank nanoparticles and the nerolidol-loaded nanoparticles. In the nerolidol-loaded nanoparticles, the low-intensity peak that belongs to the presence of nerolidol in the sample was observed following its dilution with water. This peak is most probably due to the slight release of the drug from the nanoparticles as some drug particles are weakly bonded on the nanoparticles’ surface.

### 3.3. Scanning Electron Microscope

**Figure 5:**
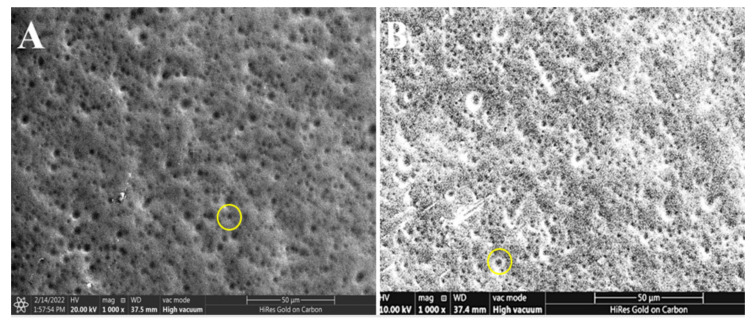
(**A**) The scanning electron microscope (SEM) image of the blank chitosan–alginate nanoparticles and (**B**) the SEM image of the nerolidol-loaded chitosan–alginate nanoparticles. The yellow circle on both figures shows the sphere-like nanoparticles morphology.

The surface morphology of the nanoparticles was examined using SEM. The images of the SEM are shown in Figure 5A,B for the blank and nerolidol-loaded chitosan–alginate nanoparticles, respectively. The images shows spherical-like particle morphology, which relates well with previously fabricated chitosan–alginate nanoparticles [26].

### 3.4. Transmission Electron Microscopy

**Figure 6:**
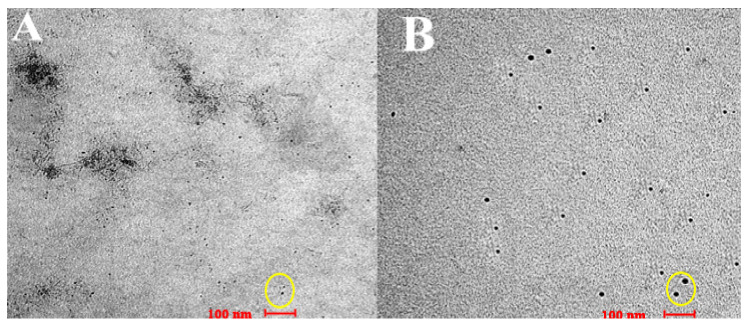
(**A**) The transmission electron microscope (TEM) image for blank chitosan–alginate nanoparticles and (**B**) the TEM image of nerolidol-loaded chitosan–alginate nanoparticles. The yellow circle on both figures shows the sphere-like nanoparticles.

The particles’ morphology was detected using TEM and the results are shown in Figure 6A,B for both blank and nerolidol-loaded chitosan–alginate nanoparticles, respectively. The nanoparticles were observed to have an almost spherical shape with some agglomerations, especially in the blank nanoparticles, and a size of approximately 30–40 nm for the loaded nanoparticles and 10–15 nm for the blank nanoparticles. The difference of size between the blank and loaded nanoparticles proves that the loading of the drug was successful.

The TEM analysis confirmed the presence of nanoparticles and provided morphological information for the blank chitosan–alginate nanoparticles and nerolidol-loaded chitosan–alginate nanoparticles close to what was previously observed in chitosan–alginate nanoparticles [26]. The nanoparticle coating plays a predominant role in protecting the incorporated active substance and in the release profile.

Currently, it is becoming more preferable to decide the size of nanoparticles using TEM imaging in a manner similar to what we have done in our manuscript. This is also based on the fact that the most accurate, precise, and high-resolution size measurement is usually obtained using electron microscopy [28]. Moreover, Mehn et al. [29] commented on this issue, stating that it is difficult to distinguish between the particle size distribution of polydispersed samples made of two or three particle populations in similar size ranges using dynamic light scattering; hence, it is difficult to distinguish between small aggregates and larger particles and to reliably measure the particle size distribution [28].

### 3.5. In Vitro Release for Oral Absorption

**Figure 7:**
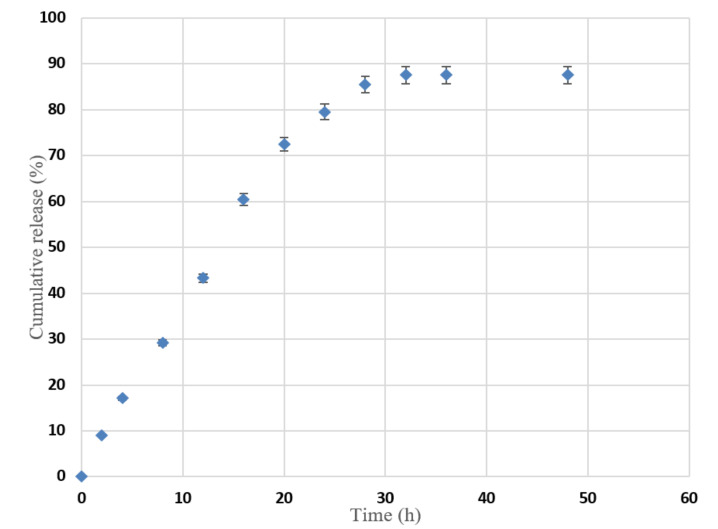
The cumulative nerolidol release as a function of time (expressed as error of standard deviation).

The release of nerolidol from the chitosan–alginate nanoparticles was found to occur in a controlled release manner over around 28 h (Figure 7) with a total release concentration and encapsulation efficiency of 87% calculated using Equation (2). This percentage accounted for 5916 µg of the initially used amount of nerolidol. Due to the volatility of nerolidol, some of the originally added drug evaporated during the preparation and characterization, so the encapsulation efficiency did not reach 100%. Using Equation (4), the nanoparticles showed a loading capacity of 51.4%. As shown in Figure 7, which was constructed using Equation (3), in the first 8 h, less than 30% of the drug was released. This initial burst was probably due to the weak forces which held some of the drug particles to the nanoparticles. Most of the release occurred between 8 and 20 h, as the remaining drug particles were probably encapsulated within the nanoparticle matrix and required extra time to be released. At 20 h and up to 28 h, the release rate declined. From 32 to 48 h, the release rate was steady with a concentration of 87%.

In a mouse model, the maximum concentration of free nerolidol achieved was found to be 0.35 µg/mL 6 h following a single oral dose of 1000 mg/kg [30], and this was twice the concentration reached in the in vitro model [30]. In our study, the maximum in vitro concentration reached was 5916 µg/mL. This rise in the released concentration compared to the release of free nerolidol was probably due to the encapsulation of most of the drug inside the nanoparticles. The encapsulation increases the water solubility of the system and eases the delivery of nerolidol to the aqueous PBS medium. Therefore, the nerolidol-loaded chitosan–alginate nanoparticles are more efficient in achieving a higher release concentration compared to the pure nerolidol.

## 4. Conclusions

In this research, we have successfully fabricated and characterized chitosan–alginate nanoparticles and loaded nerolidol into the nanoparticles. The nanoparticles were prepared by a newly modified ionic gelation method followed by crosslinking and characterization. This drug delivery system proved to be a potential carrier which caused a controlled release of nerolidol. The small size of the nanoparticles, the decent encapsulation efficiency, and the loading capacity make this system a potential carrier for other hydrophobic drugs which results in a more efficient delivery into the body.

## Data Availability

Data are available within the article.

## References

[1] Klopell F.C., Lemos M., Sousa J.P.B., Comunello E., Maistro E.L., Bastos J.K., De Andrade S.F. (2007). Nerolidol, an antiulcer constituent from the essential oil of Baccharis dracunculifolia DC (*Asteraceae*). Z. Nat.-Sect. C J. Biosci..

[2] Ferreira F.M., Palmeira C.M., Oliveira M.M., Santos D., Simões A.M., Rocha S.M., Coimbra M.A., Peixoto F. (2012). Nerolidol effects on mitochondrial and cellular energetics. Toxicol. Vitr..

[3] Parreira N.A., Magalhães L.G., Morais D.R., Caixeta S.C., De Sousa J.P.B., Bastos J.K., Cunha W.R., Silva M.L., Nanayakkara N.P., Rodrigues V. (2010). Antiprotozoal, schistosomicidal, and antimicrobial activities of the essential oil from the leaves of baccharis dracunculifolia. Chem. Biodivers..

[4] Asaikumar L., Vennila L., Akila P., Sivasangari S., Kanimozhi K., Premalatha V., Sindhu G. (2019). Preventive effect of nerolidol on isoproterenol induced myocardial damage in Wistar rats: Evidences from biochemical and histopathological studies. Drug Dev. Res..

[5] Zhang L., Sun D., Bao Y., Shi Y., Cui Y., Guo M. (2017). Nerolidol Protects Against LPS-induced Acute Kidney Injury via Inhibiting TLR4/NF-κB Signaling. Phyther. Res..

[6] Türkmen N.B., Yüce H., Çetin A., Şahin Y., Ciftci O. (2022). The Ameliorate Effects of Nerolidol on Thioasteamide-Induced Oxidative Damage in Heart and Kidney Tissue. Turk. J. Pharm. Sci..

[7] Boris R., Elena T., Erich S., Walter J., Gerhard B., Leopold J. (2011). Cytotoxic properties of selected sesquiterpene alcohols on human cervix carcinoma cell lines. J. Essent. Oil-Bearing Plants.

[8] Ryabchenkoa B., Elena Tulupovab E., Erich Schmidtc E., Wlcekd K., Buchbauerd G., Jirovetz L. (2008). Investigation of Anticancer and Antiviral Properties of Selected Aroma Samples. Nat. Prod. Commun..

[9] Prasanthi D., Lakshmi P.K. (2012). Terpenes: Effect of lipophilicity in enhancing transdermal delivery of alfuzosin hydrochloride. J. Adv. Pharm. Technol. Res..

[10] Cornwell P.A., Barry B.W. (1994). Sesquiterpene Components of Volatile Oils as Skin Penetration Enhancers for the Hydrophilic Permeant 5-Fluorouracil. J. Pharm. Pharmacol..

[11] Pinheiro B.G., Silva A.S.B., Souza G.E.P., Figueiredo J.G., Cunha F.Q., Lahlou S., Da Silva J.K., Maia J.G., Sousa P.J. (2011). Chemical composition, antinociceptive and anti-inflammatory effects in rodents of the essential oil of Peperomia serpens (Sw.) Loud. J. Ethnopharmacol..

[12] Lima D.K.S., Ballico L.J., Rocha Lapa F., Gonçalves H.P., De Souza L.M., Iacomini M., de Paula Werner M.F., Baggio C.H., Pereira I.T., da Silva L.M. (2012). Evaluation of the antinociceptive, anti-inflammatory and gastric antiulcer activities of the essential oil from Piper aleyreanum C.DC in rodents. J. Ethnopharmacol..

[13] Nibret E., Wink M. (2010). Trypanocidal and antileukaemic effects of the essential oils of Hagenia abyssinica, Leonotis ocymifolia, Moringa stenopetala, and their main individual constituents. Phytomedicine.

[14] Hoet S., Stévigny C., Hérent M.F., Quetin-Leclercq J. (2006). Antitrypanosomal compounds from the leaf essential oil of Strychnos spinosa. Planta Med..

[15] Lee S.J., Han J.I., Lee G.S., Park M.J., Choi I.G., Na K.J., Jeung E.B. (2007). Antifungal effect of eugenol and nerolidol against Microsporum gypseum in a guinea pig model. Biol. Pharm. Bull..

[16] Curvelo J.A.R., Marques A.M., Barreto A.L.S., Romanos M.T.V., Portela M.B., Kaplan M.A.C., Soares R.M. (2014). A novel nerolidol-rich essential oil from Piper claussenianum modulates Candida albicans biofilm. J. Med. Microbiol..

[17] Goulart H.R., Kimura E.A., Peres V.J., Couto A.S., Duarte F.A.A., Katzin A.M. (2004). Terpenes arrest parasite development and inhibit biosynthesis of isoprenoids in Plasmodium falciparum. Antimicrob. Agents Chemother..

[18] Lopes N.P., Kato M.J., Andrade E.H.D.A., Maia J.G.S., Yoshida M., Planchart A.R., Katzin A.M. (1999). Antimalarial use of volatile oil from leaves of Virola surinamensis (Rol.) Warb. by Waiapi Amazon Indians. J. Ethnopharmacol..

[19] Chan W.K., Tan L.T.H., Chan K.G., Lee L.H., Goh B.H. (2016). Nerolidol: A sesquiterpene alcohol with multi-faceted pharmacological and biological activities. Molecules.

[20] Li P., Dai Y.N., Zhang J.P., Wang A.Q., Wei Q. (2008). Chitosan-alginate nanoparticles as a novel drug delivery system for nifedipine. Int. J. Biomed. Sci..

[21] Makamh M. (2017). Nanotechnology Based Approach to Enhance the Potential of Chemopreventive Agent Berberine Hydrochloride in Cancer Therapy. Int. J. Biol. Pharm. Allied Sci..

[22] Sachan N., Pushkar S., Jha A., Bhattcharya A. (2009). Sodium alginate: The wonder polymer for controlled drug delivery. J. Pharm. Res..

[23] Shu X.Z., Zhu K.J., Song W. (2001). Novel pH-sensitive citrate cross-linked chitosan film for drug-controlled release. Int. J. Pharm..

[24] Lertsutthiwong P., Rojsitthisak P., Nimmannit U. (2009). Preparation of turmeric oil-loaded chitosan-alginate biopolymeric nanocapsules. Mater. Sci. Eng. C.

[25] Setty C.M., Sahoo S.S., Sa B. (2005). Alginate-coated alginate-polyethyleneimine beads for prolonged release of furosemide in simulated intestinal fluid. Drug Dev. Ind. Pharm..

[26] Katuwavila N.P., Perera A.D.L.C., Samarakoon S.R., Soysa P., Karunaratne V., Amaratunga G.A.J., Karunaratne D. (2016). Chitosan-Alginate Nanoparticle System Efficiently Delivers Doxorubicin to MCF-7 Cells. J. Nanomater..

[27] Thwala L.N. (2012). Preparation and Characterization of Alginate-Chitosan Nanoparticles as a Drug Delivery System for Lipophilic Compounds. https://ujcontent.uj.ac.za/vital/access/services/Download/uj:2752/CONTENT1.

[28] Parupudi A., Mulagapati A.H.R., Subramony J.A., Kesharwani P., Singh K. (2022). Nanoparticle technologies: Recent state of the art and emerging opportunities. Nanoparticle Therapeutics: Production Technologies, Types of Nanoparticles, and Regulatory Aspects.

[29] Mehn D., Caputo F., Rösslein M., Calzolai L., Saint-Antonin F., Courant T., Wick P., Gilliland D. (2017). Larger or more? Nanoparticle characterisation methods for recognition of dimers. RSC Adv..

[30] Saito A.Y., Sussmann R.A.C., Kimura E.A., Cassera M.B., Katzin A.M. (2015). Quantification of nerolidol in mouse plasma using gas chromatography-mass spectrometry. J. Pharm. Biomed. Anal..

